# Teicoplanin associated gene *tcaA* inactivation increases persister cell formation in *Staphylococcus aureus*

**DOI:** 10.3389/fmicb.2023.1241995

**Published:** 2023-10-13

**Authors:** Gul Habib, Haji Gul, Prevez Ahmad, Azam Hayat, Mujaddad Ur Rehman, Ihab Mohamed Moussa, Hosam O. Elansary

**Affiliations:** ^1^Department of Microbiology, Abbottabad University of Science and Technology, Abbottabad, Pakistan; ^2^College of Animal Science and Technology, Anhui Agricultural University, Hefei, China; ^3^Faculty of Veterinary and Animal Sciences, Gomal University, Dera Ismail Khan, Pakistan; ^4^Department of Physics, University of Azad Jammu and Kashmir, Muzaffarabad, Pakistan; ^5^Department of Botany and Microbiology, College of Science, King Saud University, Riyadh, Saudi Arabia; ^6^Plant Production Department, College of Food & Agriculture Sciences, King Saud University, Riyadh, Saudi Arabia

**Keywords:** MRSA, *tcaA*, *tcaB*, persister cell, glycopeptides resistance, *glyS*

## Abstract

*Staphylococcus aureus* is part of normal human flora and is widely associated with hospital-acquired bacteremia. *S. aureus* has shown a diverse array of resistance to environmental stresses and antibiotics. Methicillin-resistant *S. aureus* (MRSA) is on the high priority list of new antibiotics discovery and glycopeptides are considered the last drug of choice against MRSA. *S. aureus* has developed resistance against glycopeptides and the emergence of vancomycin-intermediate-resistant, vancomycin-resistant, and teicoplanin-resistant strains is globally reported. Teicoplanin-associated genes tcaR-tcaA-tcaB (tcaRAB) is known as the *S. aureus* glycopeptide resistance operon that is associated with glycopeptide resistance. Here, for the first time, the role of tcaRAB in *S. aureus* persister cells formation, and ΔtcaA dependent persisters’ ability to resuscitate the bacterial population was explored. We recovered a clinical strain of MRSA from a COVID-19 patient which showed a high level of resistance to teicoplanin, vancomycin, and methicillin. Whole genome RNA sequencing revealed that the tcaRAB operon expression was altered followed by high expression of *glyS* and *sgtB*. The RNA-seq data revealed a significant decrease in *tcaA* (*p* = 0.008) and *tcaB* (*p* = 0.04) expression while *tcaR* was not significantly altered. We knocked down *tcaA*, *tcaB*, and *tcaR* using CRISPR-dCas9 and the results showed that when *tcaA* was suppressed by dCas9, a significant increase was witnessed in persister cells while *tcaB* suppression did not induce persistence. The results were further evaluated by creating a *tcaA* mutant that showed ΔtcaA formed a significant increase in persisters in comparison to the wild type. Based on our findings, we concluded that *tcaA* is the gene that increases persister cells and glycopeptide resistance and could be a potential therapeutic target in *S. aureus*.

## 1. Introduction

*Staphylococcus aureus* has two major types of resistant strains, namely, methicillin-resistant *S. aureus* (MRSA) and vancomycin-resistant *S. aureus* (VRSA). MRSA is considered the major cause of hospital and community-acquired infections (Aqib and Rodriguez-Morales, 2021) and is largely treated with glycopeptides. However, the emergence of teicoplanin resistance, vancomycin-intermediate *S. aureus* (VISA), and VRSA has made the clinical treatment unsuccessful and increased death rates across the world (Howden et al., 2010). *S. aureus* resistant to glycopeptides are a serious threat to public health and teicoplanin-resistant (Elsaghier et al., 2002; Szymanek-Majchrzak et al., 2018), VISA, and VRSA strains are globally reported (Appelbaum, 2006; Shariati et al., 2020). A meta-analysis of 155 articles from 2010 to 2019 reported a global prevalence of VRSA of 2.4 and 4.3% for VISA (Shariati et al., 2020), whereas a similar study reported a 7% frequency of VRSA from 2015 to 2020, with a prevalence of 16% in Africa, 5% in Asia, and 4% in America (Wu et al., 2021). Different genes were reported for glycopeptide resistance in *S. aureus* such as the *vanA*, *yycF*, *yycG*, *tcaA*, and *ccpA* (Renzoni et al., 2009). Other include mutations in accessory gene regulator, *vraSR*, and *graSR* two-component regulatory systems (Howden et al., 2008; Hu et al., 2016). Likewise, mutations in *sigB* and *trfAB* genes also contributed to glycopeptide resistance (Renzoni et al., 2009; Schulthess et al., 2009). According to the Clinical and Laboratory Standards Institute (CLSI) guidelines, *S. aureus* strains with a minimum inhibitory concentration (MIC) of ≤2 μg/mL would be considered susceptible (Clinical and Laboratory Standards Institute, 2018) and MRSA isolates with a glycopeptide MIC of greater than 2 μg/mL reflect poor clinical outcomes (Chen et al., 2013; Song et al., 2017). Several studies documented that teicoplanin resistance developed earlier than vancomycin resistance (Brunet et al., 1990; Hiramatsu, 2001) and was related to teicoplanin resistance operon tcaRAB (Brandenberger et al., 2000; Maki et al., 2004). A study revealed that teicoplanin resistance resulted in a slight increase in vancomycin resistance (Hiramatsu, 2001). Furthermore, penicillin binding protein-2 (pbp2) ectopic expression was associated with an increase in vancomycin MIC from 1 to 2 μg/mL and teicoplanin MIC from 2 to 8 μg/mL (Sieradzki et al., 1998). Interestingly, both Gram-negative and Gram-positive bacteria use different strategies to evade antibiotic actions. One of the mechanisms bacteria have adopted is the formation of persister cells. Persisters are nongrowing or metabolically less active cells that can survive high antibiotic concentrations without being resistant (Lewis, 2010; Personnic et al., 2023). Upon favorable conditions, persisters become metabolically active, regain their virulence potential, and resuscitate the whole population that remains fully susceptible to antibiotics (Eisenreich et al., 2021). Persistence should not be confused with tolerance and resistance because tolerance is the ability of a bacterial population to survive a transient exposure to antibiotics usually higher than the MIC (Handwerger and Tomasz, 1985), whereas resistance is an inherited trait that develops due to genetic changes in bacteria (Blair et al., 2015). Resistance to antibiotics is quantified by MIC testing and substantially higher than the MIC for a susceptible strain of bacteria, whereas the MIC for a tolerant strain is similar to the susceptible strain. Similarly, a persistent strain has a similar MIC and minimum duration for killing (MDK99) to a susceptible strain, whereas the MDK99 for a tolerant strain is substantially higher than the MDK99 for a susceptible strain. However, the MDK for 99.99% (MDK99.99) of bacterial cells in the population is substantially higher for a persistent strain than the MDK99.99 for a susceptible strain (Brauner et al., 2016). Several factors have been reported in different bacteria that contributed to persister cell formation, including nutrient starvation, acidic pH, accumulation of insoluble proteins, ATP depletion, and antibiotics exposure (Levin et al., 2017; Mohiuddin et al., 2021). Particularly, ATP depletion has induced persister cell formation in *S. aureus*, *E. coli*, and *P. aeruginosa* (Cameron et al., 2018). *S. aureus purB* and *purM* mutants showed defective persistence in low pH, heat stress, and through rifampicin treatment (Lin et al., 2020). The work of Wang et al. (2015) revealed the results of the transposon mutant library of a clinical MRSA strain where gene mutations in the tricarboxylic acid cycle, oxidative phosphorylation, and ABC transporters showed a lower number of persisters. For instance, the mutant of succinate dehydrogenase was defective in persister cells formation against levofloxacin (Wang et al., 2015). Shang and coworkers inactivated *phoU* which decreased vancomycin and levofloxacin persisters (Shang et al., 2020). Tricarboxylic acid cycle genes increased persister cell formation due to reduced ATP level and membrane potential (Wang et al., 2018). Likewise, *S. aureus* grown in polymicrobial cultures displayed increase antibiotic tolerance, accompanied by low intracellular ATP and membrane potential (Jia et al., 2013). Of note, persister cells can facilitate the evolution of drug resistance because tolerance precedes resistance and can boost the chances for resistance mutations in bacterial populations (Levin-Reisman et al., 2017). Due to the COVID-19 pandemic, numerous bacterial resistant strains were reported worldwide and were associated with antibiotic resistance and treatment failure (Ghanizadeh et al., 2021; Habib et al., 2022, 2023; Tariq et al., 2023). In this study, we assessed teicoplanin- and vancomycin-intermediate-resistant *S. aureus* recovered from a COVID-19 patient and explored the involvement of tcaABR operon in glycopeptide resistance and persistence. The role of *tcaA* in glycopeptide resistance has been widely studied but *tcaA* involvement in persister cell formation has not been discovered. Using CRISPR-dCas-9, the genetic basis was explored that revealed the teicoplanin resistance gene *tcaA* was associated with an increase in persister cell formation. The inactivation of *tcaA* has shown persistence and resistance to glycopeptides whereas *tcaB* and *tcaR* have no significant effects.

## 2. Materials and methodology

### 2.1. Bacterial strains, media, vectors, and growth parameters

The *S. aureus* teicoplanin- and vancomycin-intermediate-resistant clinical strain, hereafter referred to as wild-type (WT), was obtained from Lady Reading Hospital Peshawar. Tryptic soy broth (TSB), Luria Bertani broth (LB), and cation-adjusted Mueller Hinton broth (MHB) were used for *S. aureus*, *E. coli*, and MIC testing, respectively. Bacterial cultures were refreshed from −80°C and were grown in TSB (*S. aureus*) and LB (*E. coli*) at 37°C with shaking at 220 rpm. Bacterial strains were grown in round bottom tubes (14 mL tube with 3 mL culture media) and persister assays were performed in a conical flask (100 mL flask with 30 mL culture media). Cells were washed with ddH_2_O. Temperature-sensitive (at 30°C) vector pBTs were used for mutant construction in *S. aureus*, pRMC-2 as an inducible vector for gene expression, and pALC with green fluorescent protein (GFP) gene was used for fluorescence assay. For plasmid maintenance, media were supplemented with appropriate antibiotic concentration, i.e., ampicillin 150 μg/mL and chloramphenicol 20 μg/mL. The pSD1 is a pRMC2 derivative vector that contains dCas9 and sgRNA expression cassettes. *S. aureus* RN4220 and *E. coli* DH5α were used for transformation. The primers, plasmids, recombinant vectors, and bacterial strains used in this study are listed in Table 1.

**Table 1:** The list of primers, plasmids, and bacterial strains used in this study.

Strain	Relevant genotype	Source
*S. aureus* strains		
MRSA MW2	Methicillin-resistant *S. aureus* strain MW2	NARSA
*S. aureus* WT	Clinical isolate, glycopeptide intermediate resistant strain	Hospital source
RN4220	*S. aureus* restriction modification deficient strain	NARSA
∆tcaA	*S. aureus* WT *tcaA* mutant strain	This study
*E. coli* DH5α	Transformation	TransGen
Plasmids		
pRMC2	ATC inducible plasmid, Amp ^r^ Chl ^r^	Corrigan and Foster (2009)
pBT2	Temperature sensitive (30°C) plasmid for knockout	Brückner (1997)
pBTs	Modified pBT2 plasmid for knockout, Amp ^r^ Chl ^r^	Hu et al. (2015)
pBTs-tcaA	*TcaA* mutant vector with 1,400 bp fragment	This study
pALC	pALC1484 derivative, harboring ORF of GFP and the promoter of the S10 ribosomal gene, Amp ^r^ Chl ^r^	Bao et al. (2013)
WTpRMC-tcaA	pRMC2 derivative, with ORF of tcaA, Amp ^r^ Chl ^r^	This study
pSD1	pRMC2 derivative with dCas9 and sgRNA expression cassette	Zhao et al. (2017)
dCas9-tcaA	pSD1 with sgRNA1 targeting tcaA	This study
dCas9-tcaB	pSD1 with sgRNA2 targeting tcaB	This study
dCas9-tcaR	pSD1 with sgRNA3 targeting tcaR	This study
WT-dCas9	pSD1 with non-specific sgRNA	This study
Primer name	Oligonucleotide (5′-3′)	Application
PBts-tcaA-F-KpnI	GCGGGTACCAGAGCAGTTTATAAATAACG	tcaA knockout
PBts-tcaA -R-0	TGTACAGATATGTACACAATGGTGATAAGATTACCGCAAC	tcaA knockout
PBts-tcaA -F-0	GTTGCGGTAATCTTATCACCTTGTGTACATATCTGTACAT	tcaA knockout
PBts-tcaA-R-SacI	GCGGAGCTCAGAAATCTTAATGCAACCAT	tcaA knockout
pRMC-tcaA-F-KpnI	GCGGGTACCAGGTGAAAGTATGAAATC	tcaA complementation
pRMC-tcaA-R-SacI	GCGGAGCTCCTATTTTTCTGATGTCTTG	tcaA complementation
RT-tcaA-F	GCGAAAGTATACATTAAC	qRT-PCR
RT-tcaA-R	GATATAACGCGTGCCATT	qRT-PCR
tcaA-oligo1	CTAAGAAACTTCAATTCACCTGAAGCGC	SgRNA1
tcaA-oligo2	AACGCGCTTCAGGTGAATTGAAGTTTCT	sgRNA1
tcaB-oligo3	CTAGGTAATTTGTTTGCTGGTCCAATTTC	sgRNA2
tcaB-oligo4	AACGAAATTGGACCAGCAAACAAATTACC	sgRNA2
tcaR-oligo5	CTAAGAGCAGTTTATAAATAACGTTAAC	sgRNA3
tcaR-oligo6	AACGTTAACGTTATTTATAAACTGCTCT	sgRNA3
RT-F-tcaB	CCTGGATTACCAGATATTAG	qRT-PCR
RT-R-tcaB	AAACAATACCTAAACTTGCT	qRT-PCR
RT-F-tcaR	AACTGCAAAAATGTTGAAAG	qRT-PCR
RT-R-tcaR	TTAACTAATTTAGCATCGAT	qRT-PCR
RT-F-clpP	CTAGGAGACATCAGTGAA	qRT-PCR
RT-R-clpP	CACTCATAGCGATAACAC	qRT-PCR
RT-F-gylS	ACAGAGGTTTTGTGTTCCC	qRT-PCR
RT-R-gylS	ATACTTTTGGATTCATTAAG	qRT-PCR
RT-F-sgtB	GCGATAGGTACTCAAACTCA	qRT-PCR
RT-R-sgtB	GATACCAATAAACAATGCG	qRT-PCR
RT-F-vWbp	AGAAGACTTAGAAACCAT	qRT-PCR
RT-R-vWbp	TGATTCATCACTTTTTGCTG	qRT-PCR
RT-F-ddl	GTGCAGAACACGAAGTATCG	qRT-PCR
RT-R-ddl	TGTGAAATCTCAAGCGCCTC	qRT-PCR
RT-F-Cro	GGCATTTCGATTTCTGATA	qRT-PCR
RT-R-Cro	GTGGTAATTCTAACACTTCA	qRT-PCR
RT-F-essC	ACAGGCAGATGATTACAA	qRT-PCR
RT-R-essC	CGCCATATCACTGTATTG	qRT-PCR
RT-F-EsxB	ATGGGTGGATATAAAGGTA	qRT-PCR
RT-R-EsxB	TGCCATAATGAGTAACAC	qRT-PCR
RT-F-guaA	ACTTTGGTAGCCAATACAAC	qRT-PCR
RT-R-guaA	CCGGATCAATTGTAAATG	qRT-PCR
RT-F-EsxA	TCCAGAGGAAATCAGAGCAA	qRT-PCR
RT-R-EsxA	GGACTAAGTTGTTGGAATTGC	qRT-PCR
RT-F-FtsL	CCAACCATATGACGAACAAG	qRT-PCR
RT-R-FtsL	CATAGCAATTACAGTAATC	qRT-PCR
RT-F-alr2	GCTGTAACTCAGTTTATCCA	qRT-PCR
RT-R-alr2	GTAATATGTCAACGACGGC	qRT-PCR

### 2.2. Whole genome RNA sequencing procedure

We performed RNA sequencing to study the transcriptome of the resistant isolate. *S. aureus* 24 h culture was 100 times diluted and was grown for 2 h (OD_600_ = 2) in TSB medium, cells were harvested, washed, and dissolved in RNAiso Plus (Takara Tokyo, Japan) and preserved at −80°C. RNA sequencing was performed in two biological replicates by the core sequence facility of the Institute of Microbiology Chinese Academy of Sciences. A NanoPhotometer® (Implen CA, USA), the Qubit® RNA Assay Kit and Fluorometer (Life Technologies CA, USA), and the RNA Nano 6,000 Assay Kit from the Agilent Bioanalyzer 2,100 system were used to measure RNA purity, concentration, and integrity, respectively. RNA degradation or contamination was checked by agarose gel (1%).

#### 2.2.1. Library construction for transcriptome sequencing

The NEBNext®UltraTM RNA Library Prep Kit from Illumina®(USA) was used for library preparation, and index codes were added to attribute sequences to each sample. Briefly, poly-T oligo-attached magnetic beads were used to purify mRNA from total RNA, then fragmentation was performed using divalent cations under high temperature in NEBNext First-Strand Synthesis Reaction Buffer (5X). Next, the first-strand cDNA strand was synthesized by M-MuLV Reverse Transcriptase and random hexamer primer, whereas the second-strand cDNA strand was synthesized by DNA Polymerase I and RNase H, and the ends of the remaining overhang were converted into blunt ends by the polymerase activity. Further, adenylation of 3′ ends of DNA fragments was performed, and an adaptor (NEBNext) with a hairpin loop structure was ligated, and cDNA fragments of 200-250 bp were preferentially purified by the AMPure XP system (Beckman Coulter, Beverly, USA). The size-selected, adaptor-ligated cDNA was treated with 3 μL USER Enzyme (NEB, USA) at 37°C for 15–20 min and followed by 5 min at 95°C. Next, PCR was performed with Phusion High-Fidelity DNA polymerase, Index (X) Primer, and Universal PCR primers. The PCR product was purified by the AMPure XP system. The Agilent Bioanalyzer 2,100 system was used to assess the quality of the constructed library (Wang Q. et al., 2017).

#### 2.2.2. Clustering and sequencing

The index-coded sample clustering was done on the Illumina cBot Cluster Generation System using TruSeq PE Cluster Kit v4-cBot-HS protocols. After cluster generation, the Illumina Hiseq 2,500 platform was used for library sequencing, and paired-end reads were generated.

#### 2.2.3. Quality assessment and comparative analysis

Raw reads (raw data) of fastq format were processed through in-house Perl scripts and the clean data was obtained by removing reads containing adapter and ploy-N. In addition, the low-quality reads from raw data were also removed, and the clean data Q20, Q30, GC-content, and sequence duplication levels were calculated. Clean data with high quality were used for all the downstream analyses. During data processing, the adaptor sequences and low-quality reads were removed, and clean reads were generated that were mapped to the reference genome sequence. The reads with a perfect match were annotated based on the reference genome. Hisat2 software was used to annotate the reads with the reference genome.

#### 2.2.4. Gene functional and differential expression analysis

Gene functions were annotated with the help of the following databases: Nt (NCBI non-redundant nucleotide sequences); Nr (NCBI non-redundant protein sequences); Pfam (Protein family); KOG/COG (Clusters of Orthologous Groups of proteins); Swiss-Prot (protein sequence database); KO (KEGG Orthologue database); GO (Gene Ontology). Differential expression analysis was performed using the DESeq R package (1.10.1). DESeq is used for determining differential expression in digital gene expression data using the model based on the negative binomial distribution (Anders and Huber, 2010). Benjamini and Hochberg’s approach was used to adjust the *p*-values to control for the false discovery rate. Genes with an adjusted *p* < 0.05 found by DESeq were assigned as differentially expressed.

#### 2.2.5. Gene Ontology (GO) enrichment analysis

For GO enrichment analysis of the differentially expressed genes (DEGs), the GOstats and topGO packages based on Wallenius non-central hypergeometric distribution were applied (Young et al., 2010).

#### 2.2.6. KEGG enrichment analysis

The KEGG database is a set of processes to map genes and proteins, etc. to molecular interaction and relation networks, and is used for large-scale molecular datasets analysis generated by genome sequencing^1^ (Kanehisa et al., 2007). We used KOBAS software to test the statistical enrichment of differential expression genes in KEGG pathways (Mao et al., 2005).

### 2.3. RNA isolation and RT-qPCR

*Staphylococcus aureus* cells were collected and dissolved in 1 mL of RNAiso Plus. The cell lysis was performed with the help of 0.1-mm silica beads in the FastPrep-24 automated system, and then the lysate was treated with DNase I to remove the remaining DNA. For reverse transcription and qPCR, the PrimeScript cDNA synthesis kit and SYBR Premix Ex Taq reagent kit (Takara Tokyo, Japan) were used, respectively. The StepOne real-time PCR system was used for RT-qPCR analysis. The *hu* gene cDNA abundance was used for normalization (Valihrach and Demnerova, 2012).

### 2.4. Gene knockdown vector pSD1

For gene knockdown, the anhydrotetracycline (ATC) inducible vector pSD1 was used which expresses dcas9 and custom-designed sgRNA. The pSD1 vector was derived from *Streptococcus pyogenes* cas9 and was used as a CRISPR interference system (CRISPRi) (Zhao et al., 2017). From the vector design, the dcas9 and sgRNA are under the control of the P_tetO_ and P_pflB_ inducible and constitutive promoters, respectively. For *tcaA*/*tcaB*/*tcaR* knockdown, gene specific complementary oligonucleotide sequences were synthesized and cloned into the SapI-digested pSD1 site. The sgRNA 5′ variable region binds to the target gene while the 3′ constant region binds to dCas9. The dCas9 functions as a DNA-binding protein guided by sgRNA which is complementary to each target gene. The pSD1 plasmid without the target gene (WT-dCas9) was used as a negative control that can also express the sgRNA which has no specific target site in *S. aureus*.

### 2.5. Construction of the *tcaA* mutant strain

To create the *tcaA* mutant, the upstream and downstream regions were amplified by primer pairs PBts-tcaA-F-KpnI and PBts-tcaA-R-0, and PBts-tcaA-F-0 and PBts-tcaA-R-SacI from *S. aureus* WT genome, respectively, (Table 1). The PBts-tcaA-R-0 and PBts-tcaA-F-0 are the overlap primers that contain the overlap segments, and a 1,400-bp fragment was generated by PBts-tcaA-F-KpnI and PBts-tcaA-R-SacI in Prime Star PCR conditions: 95°C for 5 min, 95°C for 30 s, 55°C for 1.5 min, 72°C for 1 min, 35 cycles, and 72°C for 10 min. A 1400-bp joint fragment was constructed, sequenced, and purified to clone into pBTs, a modified vector of pBT2 (Brückner, 1997; Bae and Schneewind, 2006) via restriction enzymes. The 1,400 bp fragment and pBTs vector were digested with KpnI and SacI for 1 h at 37°C, the mixture was purified by a DNA purification kit separately and ligated into pBTs by DNA ligase. The constructed recombinant vector was initially transferred to DH5α, then transferred to RN4220 for genomic modification, and lastly to *S. aureus* WT. *S. aureus* colonies carrying the recombinant vector were selected by tryptic soy agar plus chloramphenicol (20 μg/mL) at 37°C. *S. aureus* carrying the recombinant vector pBTs-tcaA was grown at 30°C with shaking at 220 rpm for 24–72 h. Daily screening was performed on ATC inducible tryptic soy agar plates at 37°C. PCR was performed to screen the mutant colonies. The *tcaA* markerless mutant was confirmed by DNA sequencing.

### 2.6. Antibiotic susceptibility and MIC testing

We followed our previous method of antibiotic susceptibility testing in *S. aureus* (Habib et al., 2020). Initially, the *tcaA* complementary strain (C-tcaA) was constructed by amplifying the *tcaA* gene through primer pairs pRMC-tcaA-F-KpnI and pRMC-tcaA-R-SacI, and the vector WTpRMC-tcaA was constructed and transferred to *S. aureus* WT. The 24 h fresh culture of *S. aureus*, ∆tcaA, and C-tcaA (complementary strain) was grown for 2 h in TSB (OD_600_ = 2.0) and 2 × 10^9^ CFU/mL were challenged with 100 fold MIC of teicoplanin, vancomycin, and azithromycin at 37°C. The inhibition zones were mapped after 24–36 h. MIC was determined in MHB medium using the two-fold broth microdilution method (Clinical and Laboratory Standards Institute, 2018). Briefly, the double dilutions of antibiotics were distributed into microtiter plates wells, and freshly prepared bacterial suspension (5 × 10^5^ CFU/mL) was added to each well, and plates were incubated at 35°C. *S. aureus* colonies suspension with a density of 5 × 10^5^ CFU/mL required a transfer of 100 μL of the 0.5 McFarland equivalent suspension to 10 mL of broth. Of note, CLSI does not recommend performing vancomycin susceptibility testing using the disk diffusion method.

### 2.7. Western blot assay

The protein expression levels of *tcaA*, *tcaB,* and *tcaR* were detected by specific antisera for *tcaA*, *tcaB*, and *tcaR* through Western blotting. The dCas9-tcaA, dCas9-tcaB, and dCas9-tcaR were induced at OD_600_ = 0.4 with ATC 100 ng/mL for 1 h. The cells were collected, and media were removed and washed twice with ddH_2_O. An equal number of cells were taken and lysed by lysostaphin for 1 h at 37°C. The mixture was washed with normal saline and total proteins were collected in lysis buffer. A 12% SDS-PAGE was used for whole-cell protein separation and proteins were electrotransferred to a polyvinylidene difluoride membrane. The tcaA, tcaB, and tcaR proteins were detected with rabbit anti-tcaA, anti-tcaB, and anti-tcaR antibodies (1:1000) followed by horseradish-peroxidase conjugated antibody (1:10,000 dilution). The membrane was analyzed using a Thermo Fisher chemiluminescent detection kit and spots were detected using an ImageQuant LAS 4000.

### 2.8. Persister assay

A persistence assay was adopted from our previous protocol (Habib et al., 2020). The cells were grown to OD_600_ = 0.40 in the TSB medium. The pSD1 containing dCas9-tcaA and dCas9-tcaB were induced with ATC 100 ng/mL for 1 h, the medium was removed, and fresh TSB and antibiotic were added whereas ΔtcaA cells were challenged with antibiotic at OD_600_ = 0.40. After 12 h, a 200 μL sample was taken and CFU counting was performed. Each antibiotic concentration was 10 fold of MIC, and *S. aureus* WT and *S. aureus* WT-dCas9 were used as controls.

### 2.9. Fluorescence microscopy

Fluorescence microscopy was used to observe the persister cell resuscitation ability of the whole population. *S. aureus* WT and ΔtcaA were transferred with pALC fluorescence shuttle plasmid with GFP and challenged with 20-fold MIC for 48 h. The cells were harvested, washed with ddH_2_O, and resuspended in TSB media. The cells were grown for 2 h at 37°C and the *S. aureus* WT and ΔtcaA were observed under the fluorescence microscope.

### 2.10. Growth curve analysis

The overnight culture was 100 times diluted in TSB medium and a 200 μL was inoculated into a 96-well microtiter plate. The plates were incubated at 37°C with shaking at 200 rpm. At different time intervals (1–18 and 24 h), OD_600_ was measured using a microplate reader (Thermo Fisher, Waltham, MA, USA).

### 2.11. Statistical analyses

Experiments were performed in biological triplicates unless otherwise stated. The data values were analyzed by Student’s *t*-test for two groups (unpaired, two-tailed) and a one-way analysis of variance for more than two groups. The gene sequences were analyzed by vector NTI advance and data were analyzed by GraphPad prism 8. * *p* < 0.05; ** *p* < 0.01; *** *p* < 0.005.

## 3. Results

### 3.1. Characterization of glycopeptide intermediate resistant *Staphylococcus aureus* (WT)

**Figure 1:**
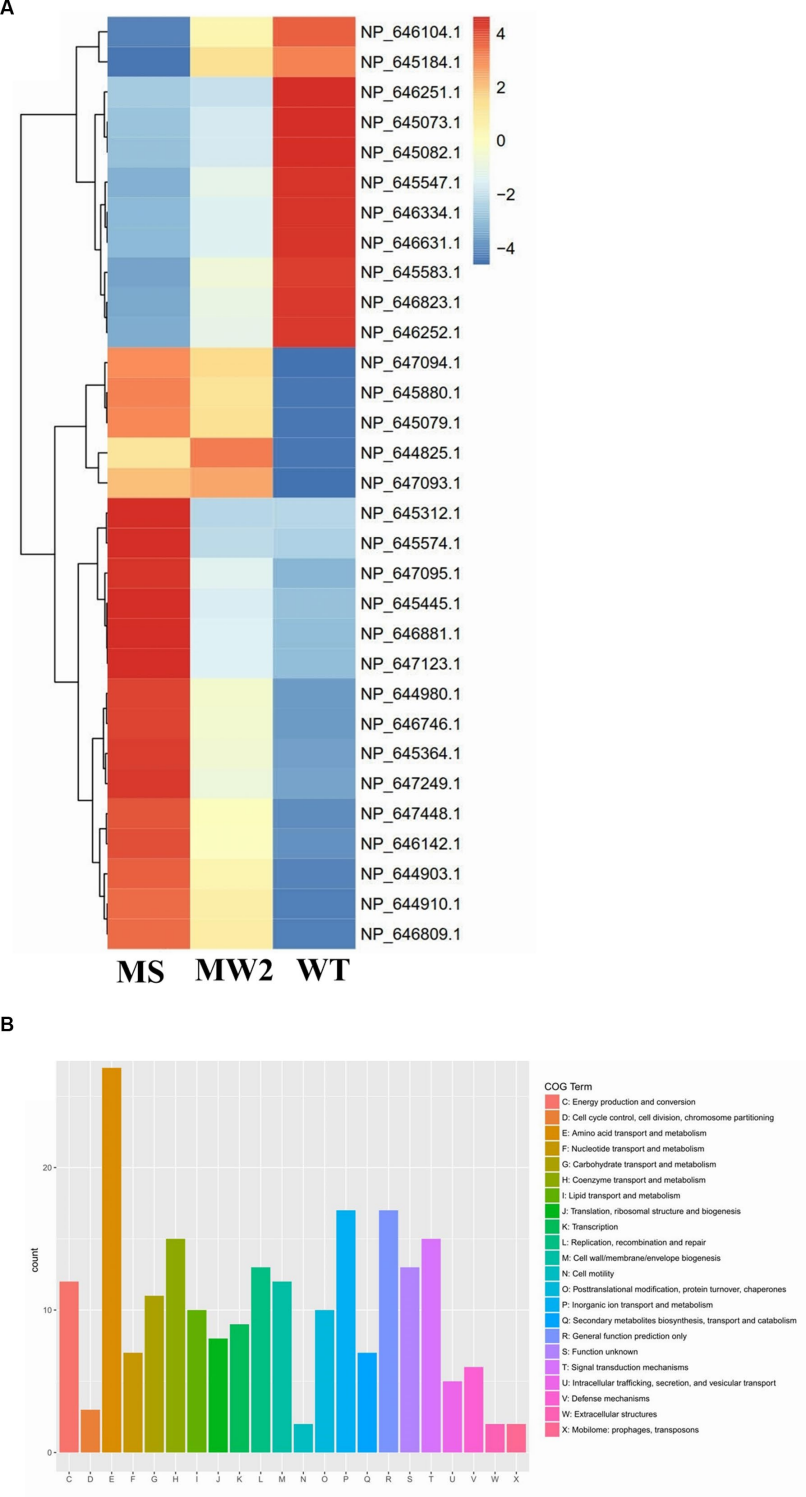
RNA-seq analysis. **(A)** The heat map of differentially expressed genes. Red represents upregulated genes and blue indicates downregulated genes. The tcaRAB operon genes *tcaA* (NP_647094.1) and *tcaB* (NP_647093.1) were significantly downregulated. The differentially expressed genes of *S. aureus* WT were compared with MS (methicillin-sensitive *S. aureus*) and MRSA MW2 as reference strains. **(B)** The COG function classification of differentially expressed genes was performed in *S. aureus* WT vs. MRSA MW2.

**Figure 2:**
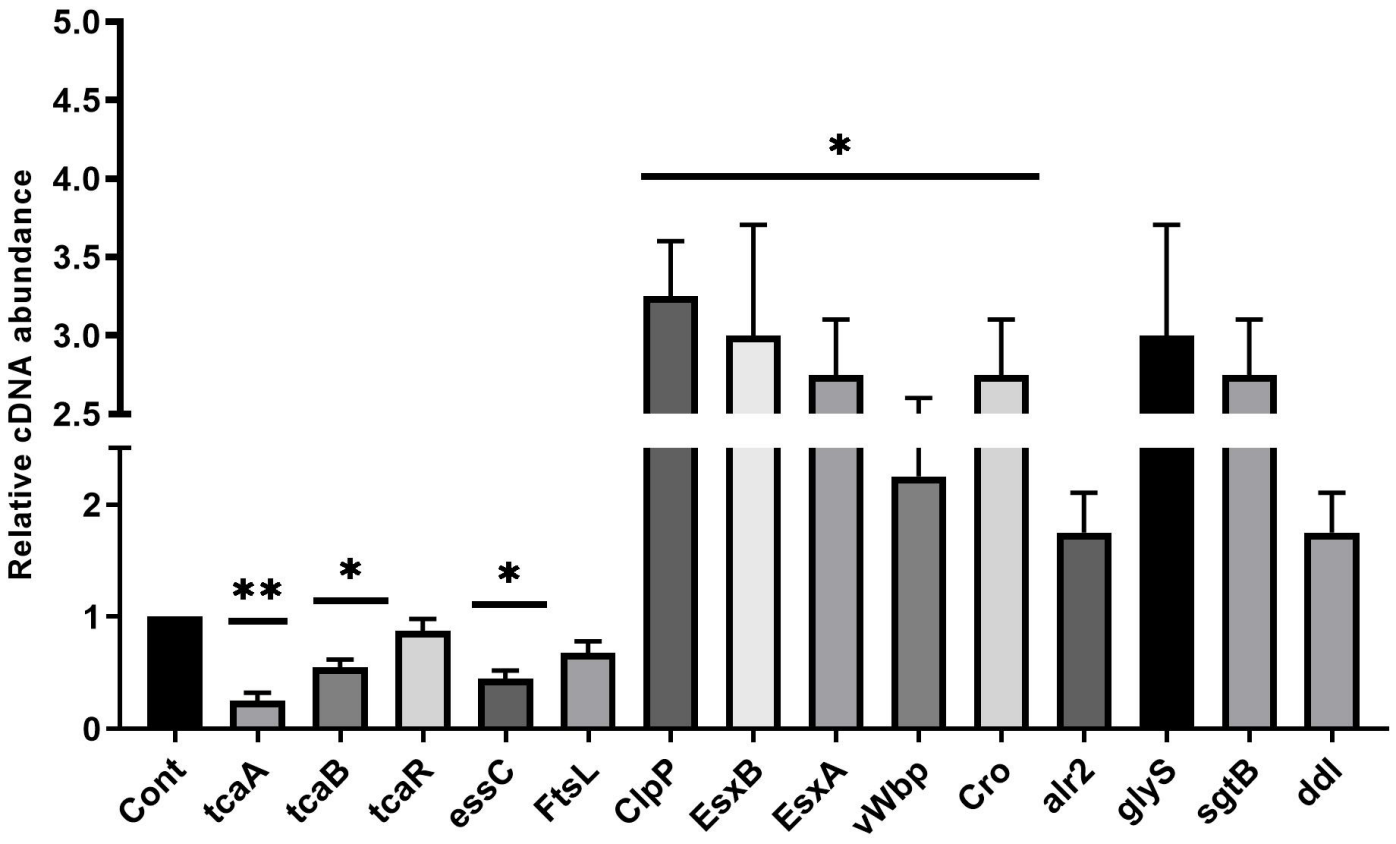
The up and downregulated genes. The RT-qPCR analysis revealed the transcript level of tcaRAB, *essC*, *FtsL*, *ClpP*, *EsxB*, *EsxA*, *glyS*, *alr2*, *sgtB*, *ddl*, *Cro*, and *vWbp* in *S. aureus* WT strain. * *p* < 0.05; ** *p* < 0.01.

*Staphylococcus aureus* isolated from a COVID-19 patient was resistant to methicillin, teicoplanin, and vancomycin, while sensitive to azithromycin and ciprofloxacin. The MIC of methicillin was >512 μg/mL, teicoplanin was 16 μg/mL, and vancomycin was 10 μg/mL (Table 2). RNA sequencing results of WT revealed that multiple genes and pathways have been affected, comprising 62 differentially expressed genes. The differential expression of highly affected genes is shown by a heat map (Figure 1A). Among the differential expressed genes, Clusters of Orthologous Genes (COG) involved in cell motility (N), extracellular structures (W), prophages and transposons (V), cell division, and chromosome partitioning (D) were marginally expressed whereas amino acid transport and metabolism (E), inorganic ion transport and metabolism (P), and general function prediction (R) were highly expressed (Figure 1B). The COG group associated with nucleotide transport and metabolism (F), translation (J), transcription (K), secondary metabolites biosynthesis (Q), intracellular trafficking (U), defense mechanism (V), cell wall/membrane/envelope biogenesis (M) were slightly expressed (Figure 1B). Differentially expressed genes such as *ClpP*, *EsxB*, *EsxA,* toxin-like hypothetical protein, *glyS*, *Cro*, *sgtB*, *ddl*, *alr2*, and *vWbp* genes expression was high whereas *tcaA*, *essC*, *tcaB*, *FtsL*, and ABC transporter permease gene expression was low (Table 3). We screened out the genes responsible for glycopeptide resistance in *S. aureus* which is reported to be the tcaRAB operon. The tcaRAB is stimulated when *S. aureus* is exposed to a minimum inhibitory concentration of glycopeptides. In the tcaRAB operon, the *tcaA* is a 454-residues zinc ribbon domain-containing protein, the *tcaB* is a 402-residues multidrug efflux MFS transporter, and *tcaR* is a 151-residues MarR family transcriptional regulator. The WT strain RNA sequencing data revealed a significant decrease in *tcaA* (*p* = 0.008) and *tcaB* (*p* = 0.04) expression while *tcaR* was not significantly altered (*p* = 0.08) (Figure 1A and Table 3). We validated the RNA sequence data by RT-qPCR which confirmed that *tcaAB* and *essC* genes were significantly suppressed whereas *EsxAB*, *ClpP*, *glyS*, and *sgtB* were significantly upregulated (Figure 2 and Table 3). The EsxAB are the secretory protein of type seven secretion system (T7SS), ClpP is involved in proteostasis, glyS is glycine tRNA synthetase, sgtB is mono-functional peptidoglycan glycosyltransferase, ddl codes for D-alanine-D-alanine ligase/synthetase, and alr2 is alanine racemase. From this data, we proposed that *EsxAB*, *ClpP*, *glyS*, and *sgtB* expression was upregulated when *tcaA* was suppressed which might be linked with cell wall protection against wall-damaging agents such as glycopeptides and viruses. Besides, the T7SS ATP synthesis machinery gene *essC* was significantly downregulated (Figure 2) which is associated with *S. aureus* survival during host infection. Collectively, these results corroborated the RNA sequencing data and confirmed that *tcaAB* suppression is associated with glycopeptides resistance either to protect the cell wall against wall-piercing agents or to increase survival during host infection.

**Table 2:** MICs of antibiotics.

Antibiotics	*S. aureus* WT	ΔtcaA	
Methicillin	≥512 μg/mL	≥256 μg/mL	
Teicoplanin	16 μg/mL	18 μg/mL	
Vancomycin	10 μg/mL	10 μg/mL	
Azithromycin	≤8 μg/mL	≤8 μg/mL	
Ciprofloxacin	≤2 μg/mL	≤2 μg/mL	

**CLSI criteria for MIC testing (μg/ml)**	**Susceptible**	**Intermediate**	**Resistance**
Vancomycin (*S. aureus*)	≤2	4–8	≥16
Teicoplanin (All Staphylococci)	≤8	16	≥32

**Table 3:** RNA-seq data of differentially expressed genes in *S. aureus* WT.

Accession number (Reference genome)	Genes / Description	*S. aureus* WT	*p*-value
NP_646251.1	Toxin like hypothetical protein	4.64 ± 0.40	0.006
NP_645073.1	T7SS *EsxA*	4.34 ± 0.32	0.007
NP_645082.1	T7SS *EsxB*	4.24 ± 0.27	0.008
NP_645547.1	*ClpP*	4.25 ± 0.20	0.006
NP_646334.1	*gylS*	4.19 ± 0.25	0.040
NP_646631.1	*sgtB*	4.16 ± 0.26	0.030
NP_645583.1	*vWbp*	4.14 ± 0.20	0.030
NP_646823.1	*ddl*	4.10 ± 0.25	0.050
NP_646252.1	*Cro*	4.06 ± 0.20	0.040
NP_646104.1	*alr2*	4.04 ± 0.20	0.050
NP_645184.1	*guaA*	4.10 ± 0.35	0.060
NP_645762.1	*Chitinase B*	4.03 ± 0.20	0.060
NP_645294.1	*ctsR*	4.02 ± 0.20	0.070
NP_644846.1	*mecA*	3.25 ± 0.45	0.120
NP_647094.1	*tcaA*	−4.53 ± 0.15	0.008
NP_645880.1	*FtsL*	−4.30 ± 0.20	0.030
NP_647093.1	*tcaB*	−4.25 ± 0.20	0.040
NP_645079.1	*essC*	−4.20 ± 0.20	0.050
NP_644825.1	ABC transporter like hypothetical protein	−4.15 ± 0.20	0.050
NP_646142.1	Facilitator transporter protein	−3.80 ± 0.30	0.050
NP_647448.1	*Ribonuclease P*	−3.70 ± 0.40	0.080
NP_644903.1	*sirA*	−4.22 ± 0.11	0.230
NP_646809.1	*MazF*	−4.17 ± 0.26	0.160
NP_644910.1	*SbnH*	−4.15 ± 0.20	0.110
NP_644980.1	*murQ*	−4.10 ± 0.30	0.250
NP_647095.1	*tcaR*	−3.00 ± 0.25	0.080

### 3.2. Knockdown of *tcaA*, *tcaB*, and *tcaR* By CRISPR-dCas9

**Figure 3:**
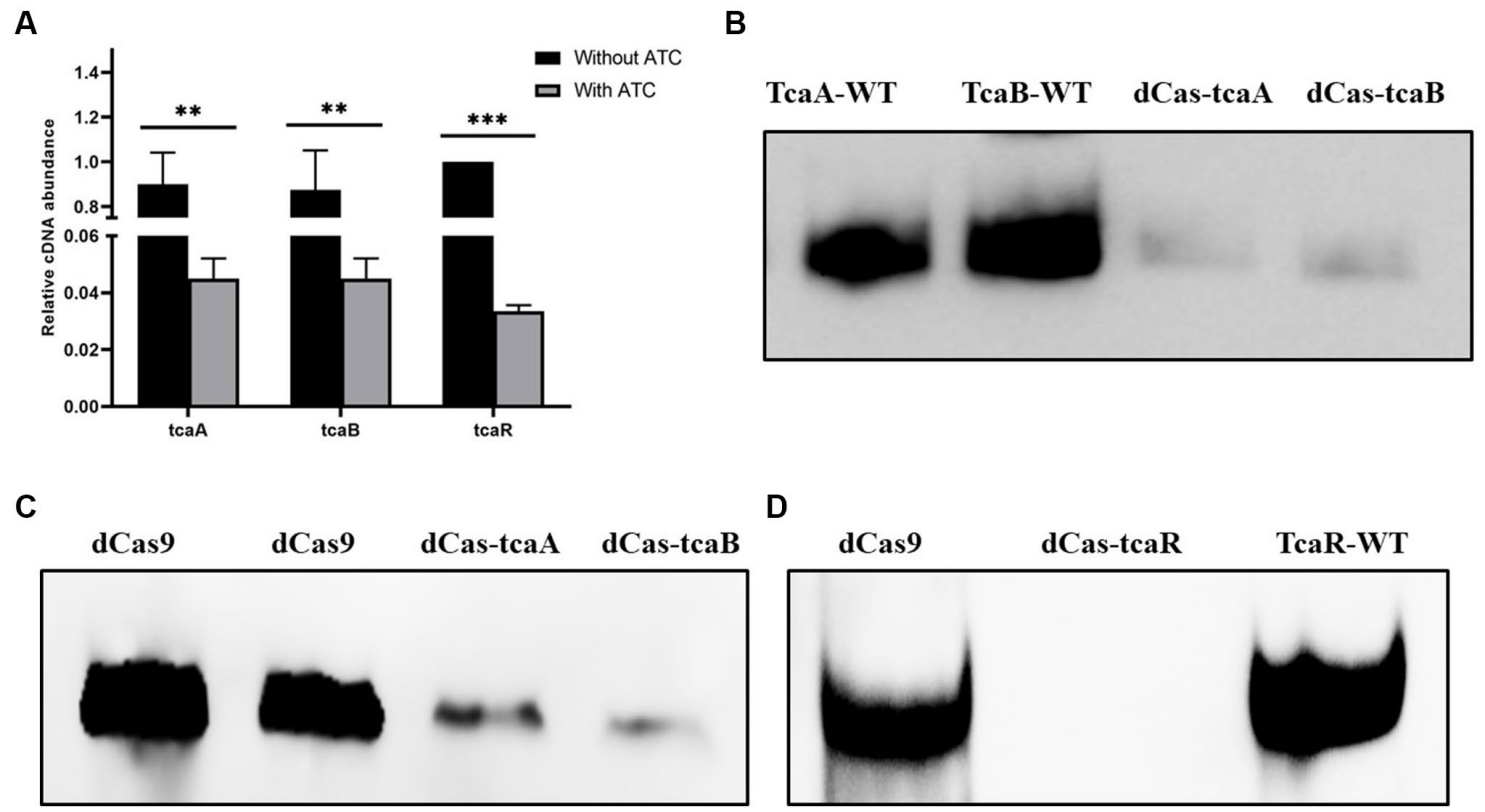
CRISPR-dCas9 mediated suppression. **(A)** The transcript level of tcaRAB operon before and after ATC induction (100 ng/mL). * *p* < 0.05, ** *p* < 0.01, *** *p* < 0.005. **(B)** The protein expression level was detected by Western blot before and after ATC induction (100 ng/mL). TcaA-WT and TcaB-WT bands are brighter, whereas dCas-TcaA and dCas-TcaB bands are dim, indicating *tcaA* and *tcaB* suppression. **(C)** The dCas9 was used as negative control (blank vector) and brighter bands were detected, while the dCas-TcaA and dCas-TcaB showed tcaA and tcaB protein suppression. **(D)** The suppression of tcaR protein was confirmed by dCas-tcaR where no protein band was detected whereas TcaR-WT and dCas9 have clear tcaR protein bands.

We performed CRISPRi-mediated *tcaA*, *tcaB*, and *tcaR* knockdown by dCas9 in *S. aureus*. Gene-specific sgRNAs were designed that can bind to the target site of *tcaA*, *tcaB*, and *tcaR* (Table 1). Media were supplied with 100 ng/mL of ATC to induce the CRISPR-dCas9 to suppress gene expression. Initially, the dCas9 repression efficiency was determined in both the knockdown and WT strain by RT-qPCR. The data revealed that the strains expressing sgRNA1, sgRNA2, and sgRNA3 exhibited a 22-fold, 20-fold, and 26-fold decrease in the *tcaA*, *tcaB*, and *tcaR* mRNA levels, respectively, (Figure 3A). Further, we analyzed the dCas-tcaA, dCas-tcaB, and dCas-tcaR protein expression level by rabbit anti-tcaA, anti-tcaB, and anti-tcaR antibodies through Western blotting. The immunoblots showed brighter tcaA, tcaB, and tcaR bands in *S. aureus* WT whereas suppression was seen in dCas-tcaA, dCas-tcaB, and dCas-tcaR carrying *S. aureus* (Figures 3B–D). The dCas9 was used as a negative control to check the inhibitory effects of the dCas-9 vector on tcaRAB proteins. From Figures 3B–D, it is confirmed that CRISPRi-mediated *tcaA*, *tcaB*, and *tcaR* knockdown was successful and can be used for persister assay.

### 3.3. The ΔtcaA increased persistence to glycopeptides antibiotics

**Figure 4:**
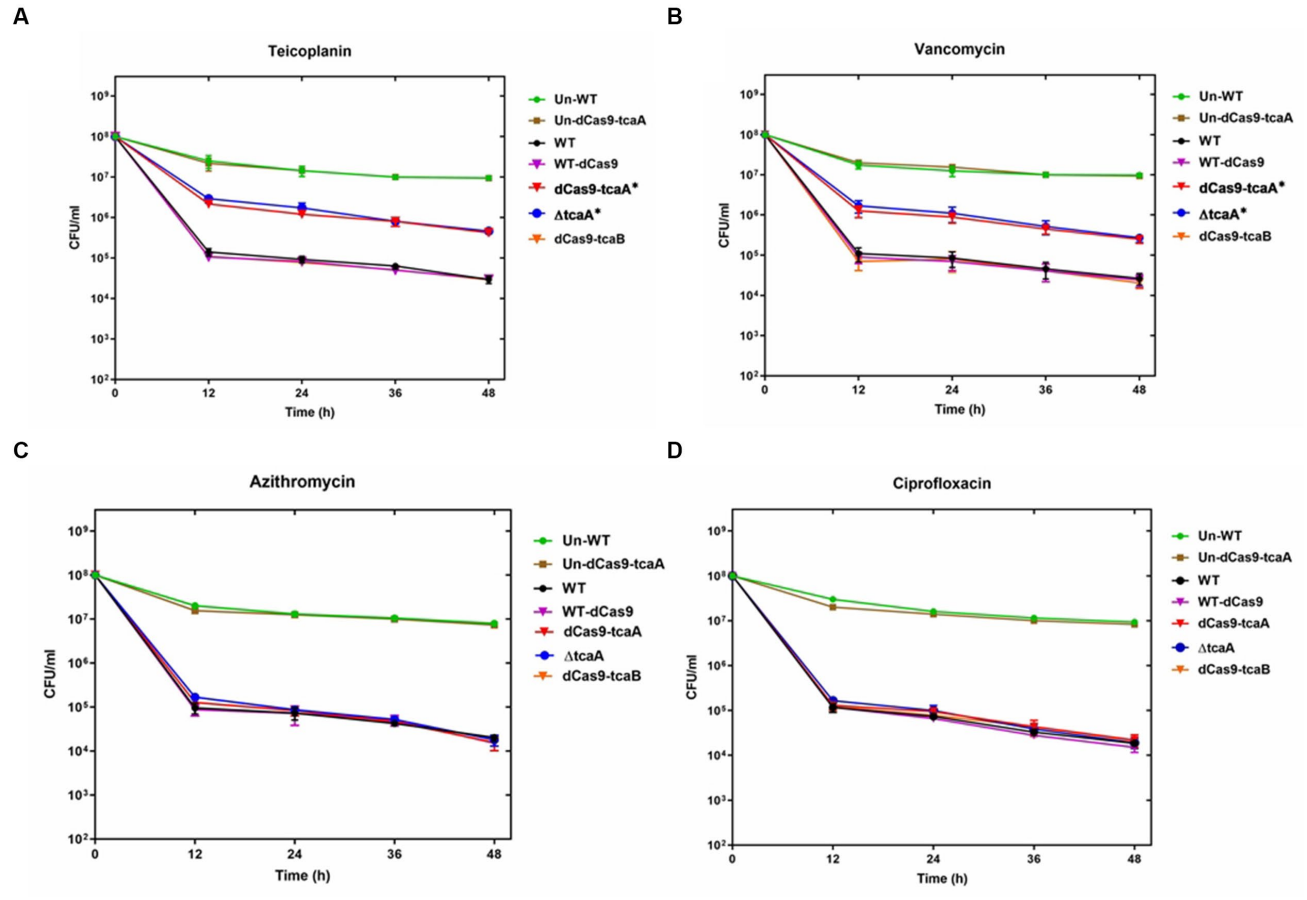
Persister assay. **(A–D)** The WT, WT-dCas9, dCas9-tcaA, dCas9-tcaB, and ΔtcaA were challenged with 10-fold MIC of teicoplanin for 48 h. The sample was taken after 12 h and CFU counting was performed. The dCas9-tcaA and ΔtcaA significantly increased the number of persister cells in panel **(A,B)**. No significant changes were observed in azithromycin and ciprofloxacin persister assays **(C,D)**. Un-WT and the Un-dCas9-tcaA were used as untreated antibiotic control strains. *S. aureus* WT and WT-dCas9 were used as controls. Experiments were performed in triplicates and error bars denote standard deviation. Statistical significance was determined using Student’s *t*-test (control versus treatment). * *p* < 0.05.

**Figure 5:**
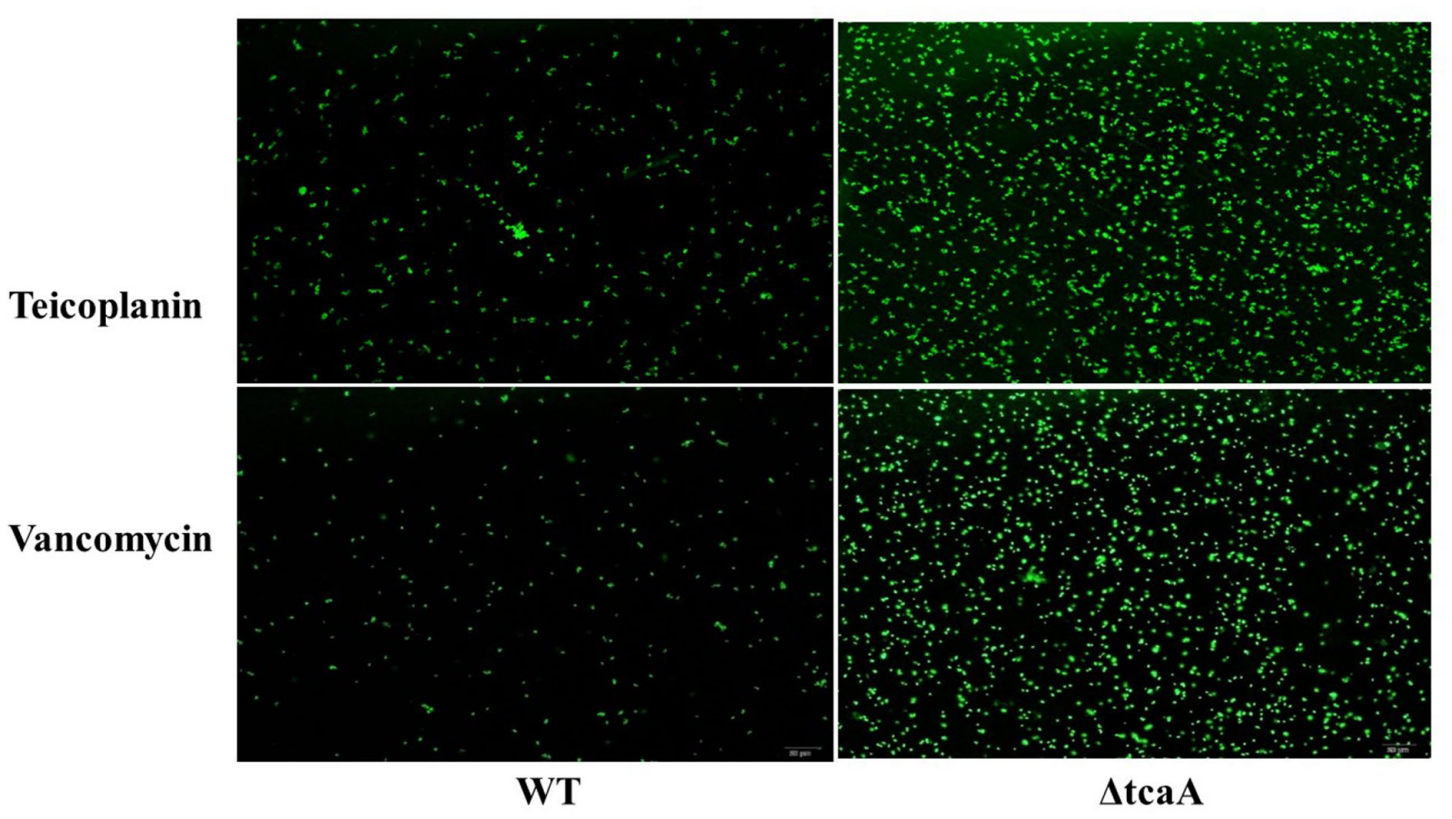
The fluorescence microscopy. *S. aureus* WT showed very dim GFP expression that revealed a low number of persisters. The ΔtcaA cells have brighter GFP expression that showed a higher number of persister and resuscitation of the bacterial population.

We performed a persistence assay with the WT, WT-dCas9, dCas9-tcaA, ΔtcaA, and dCas9-tcaB strains. The WT-dCas9 was the *S. aureus* WT strain expressing target unspecific sgRNA (control), dCas9-tcaA was the WT strain expressing sgRNA binding to *tcaA* (sgRNA1), dCas9-tcaB was WT expressing sgRNA binding to *tcaB* (sgRNA2), and ΔtcaA was *tcaA* mutant in *S. aureus* WT. All the strains were challenged with 10-fold MIC of teicoplanin, vancomycin, ciprofloxacin, and azithromycin which revealed that the dCas9-tcaA and ΔtcaA had a higher number of persisters compared to WT-dCas9 and WT strain. After 12 h of teicoplanin treatment, the surviving fraction of cells of the dCas9-tcaA and ΔtcaA strains were 17 and 18 times more than control strains WT-dCas9 and WT, respectively, (Figure 4A). Similarly, upon vancomycin treatment, the dCas9-tcaA and ΔtcaA showed a 15- and 16-fold increase relative to controls, respectively, (Figure 4B). After 48 h, the dCas9-tcaA and ΔtcaA showed a 13- and 14-fold increase in persister cells in response to teicoplanin and a 10- and 11-fold increase toward vancomycin relative to controls, respectively, (Figures 4A,B). The dCas9-tcaB did not induce persister cell formation and was similar to WT-dCas9 and WT strain (Figures 4A,B). When the cells were challenged with azithromycin and ciprofloxacin, the dCas9-tcaA, ΔtcaA, and dCas9-tcaB showed similar results with controls (Figures 4C,D). Overall, neither of the strains showed persister cell formation toward azithromycin and ciprofloxacin that confirmed the emergence of glycopeptides persisters due to *tcaA* suppression and deletion. Further, we transferred the pALC shuttle vector (with GFP) to *S. aureus* WT and ΔtcaA competent cells and challenged them with a 20-fold MIC of teicoplanin and vancomycin. After 48 h, the cells were washed and resuspended in a fresh TSB medium without antibiotics, and fluorescence microscopy was performed. The microscopy revealed a high number of cells expressing GFP in ΔtcaA cells compared to WT (Figure 5). This confirmed that persister cells tolerated a high concentration of teicoplanin and vancomycin (20 fold MIC) for 48 h and resuscitated the whole population when the antibiotic was removed. Collectively, the *tcaA* gene inactivation developed persistence to glycopeptide antibiotics while the *tcaB* did not influence the persistence phenotype. This data disclosed that *S. aureus* ΔtcaA formed persister cells and confirmed the RNA-seq and RT-qPCR results where suppression of *tcaA* was associated with the development of glycopeptides resistance. To date, Brandenberger et al. (2000) and Maki et al. (2004) reported ΔtcaA involvement in glycopeptides resistance, and the present data corroborated their results and reproduced the findings that the ΔtcaA showed resistance to teicoplanin and vancomycin and C-ΔtcaA strain restored the phenotype by expressing the *tcaA* gene via WT-pRMC-tcaA (Supplementary Figure S1).

### 3.4. The ΔtcaA growth analysis

**Figure 6:**
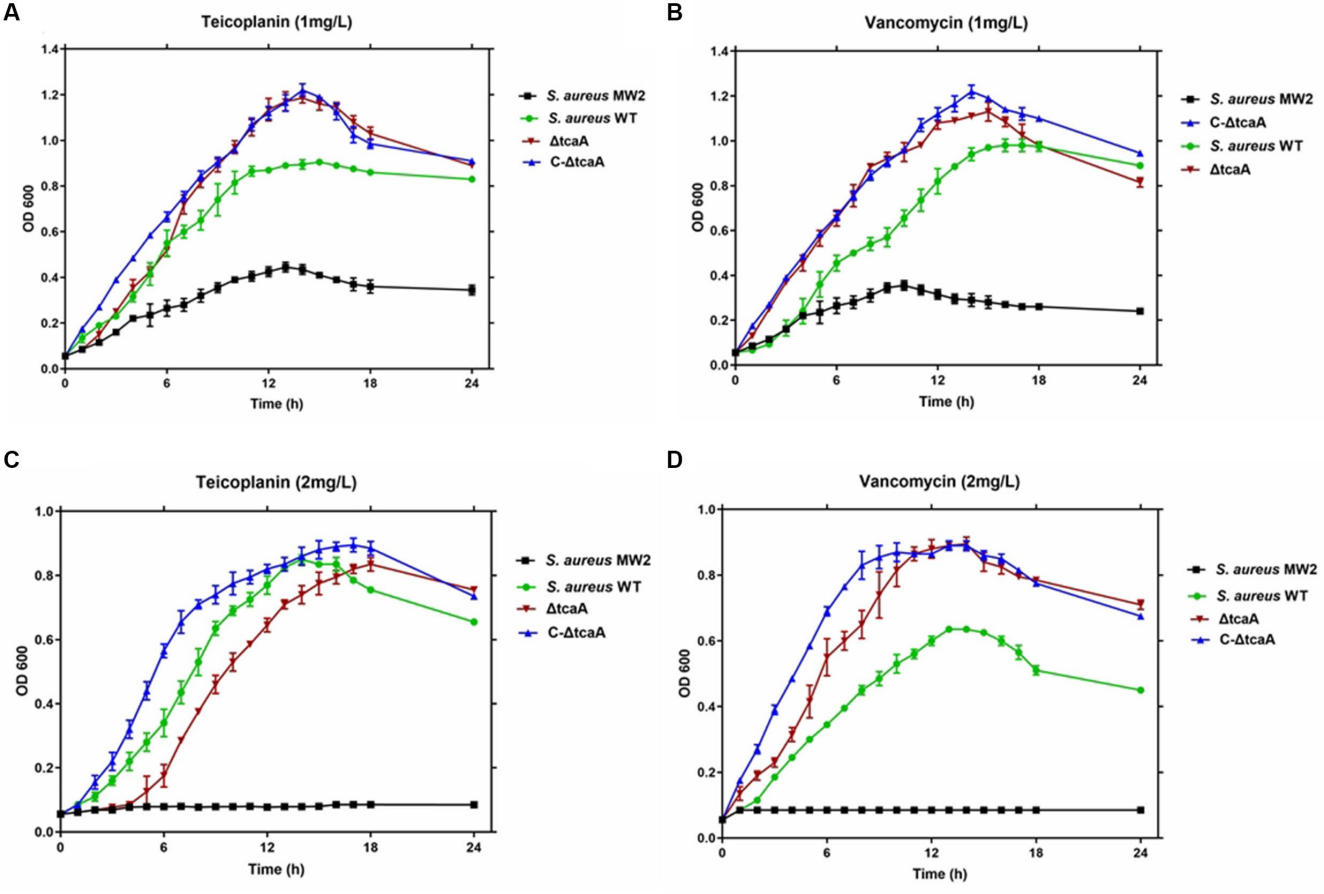
Growth curves. The *S. aureus* MW2, *S. aureus* WT, ΔtcaA, and C-ΔtcaA were grown in the TSB medium containing 1 mg/L and 2 mg/L of teicoplanin and vancomycin. **(A,B)** The growth curves at 1 mg/L of teicoplanin and vancomycin showed slow growth by MW2 and fast by *S. aureus* WT, ΔtcaA, and C-ΔtcaA. **(C,D)** At 2 mg/L of teicoplanin and vancomycin, *S. aureus* MW2 showed no growth, whereas *S. aureus* WT, ΔtcaA, and C-ΔtcaA displayed a slow growth.

We tested cell growth in the TSB medium with 1–2 mg/L of teicoplanin and vancomycin. The results showed that the MRSA MW2 reference strain displayed slow growth rates at 1 mg/L, whereas the WT, ΔtcaA, and C-ΔtcaA strains displayed fast and steady growth (Figures 6A,B). At 2 mg/L, MRSA MW2 did not show any growth whereas WT, ΔtcaA, and ΔtcaA complementary strain displayed slow growth (Figures 6C,D). These results confirmed a decrease in susceptibility of the WT, C-ΔtcaA, and ΔtcaA to teicoplanin and vancomycin compared to MW2. We conclude that *S. aureus* WT, ΔtcaA, and C-ΔtcaA strains grow at 1–2 mg/L concentration of glycopeptides which supports the fact that *tcaA* is involved in the emergence of glycopeptides resistance.

### 3.5. Inactivation of the *tcaA* influences the expression of cell wall biosynthesis genes

**Figure 7:**
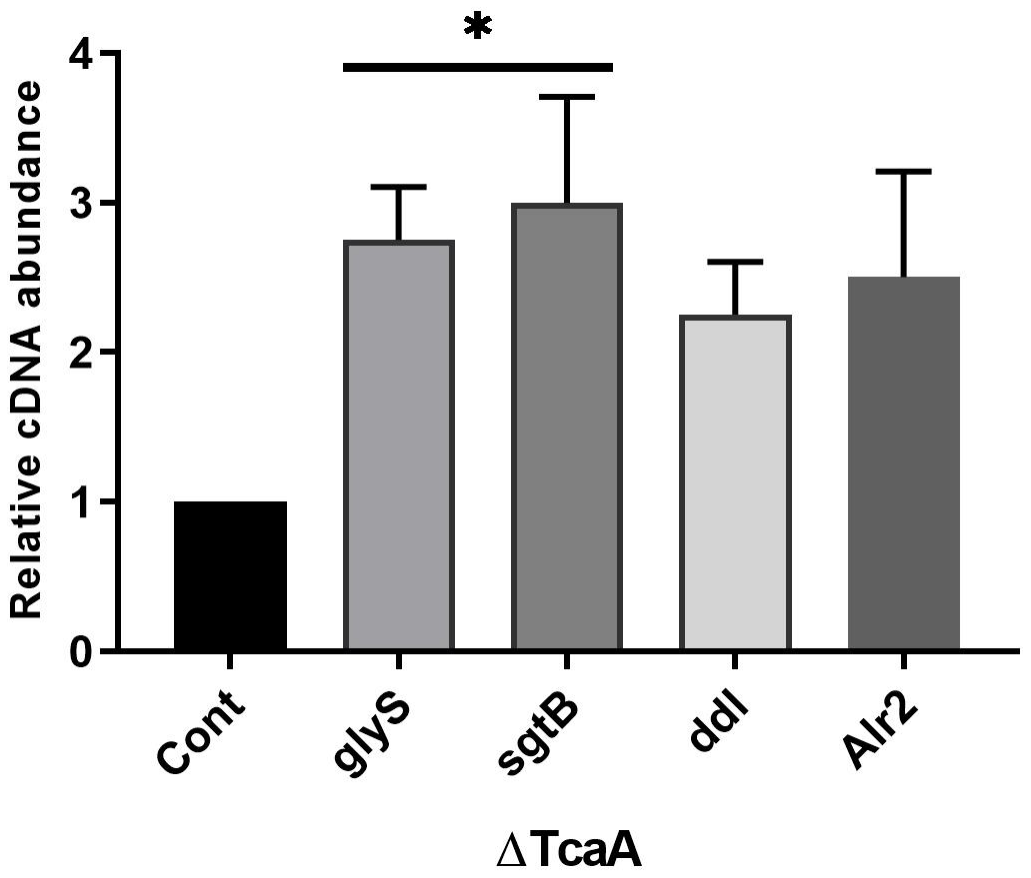
The cell wall-associated genes analysis in ΔtcaA. The *glyS* and *sgtB* showed significant expression in the mutant strain relative to *S. aureus* WT, whereas *ddl* and *Alr2* were not significant. * *p* < 0.05.

The present data indicated that *tcaA* is involved in cell wall-associated glycopeptide resistance and persistence. From RNA-seq data, the *tcaA* was significantly suppressed and *tcaA* deletion increased persister cell formation. During *tcaA* suppression, the *glyS*, *sgtB*, *ddl*, and *alr2* transcript level was high. We hypothesized that evaluating these gene expressions in ΔtcaA would validate the RNA-seq data and disclose the correlation. From RT-qPCR analysis, the *glyS* and *sgtB* expression was significant in the *tcaA* mutant while *ddl* and *alr2* transcript level was not significant (Figure 7). The high expression of *glyS* and *sgtB* might help in cell wall biogenesis because *glyS* is glycine tRNA synthetase which is involved in the supply of glycine for incorporation into nascent polypeptides during bacterial cell wall synthesis (Schneider et al., 2004; Giannouli et al., 2009) whereas *sgtB* is a mono-functional peptidoglycan glycosyltransferase which is involved in peptidoglycan synthesis and also supports the growth of *S. aureus* in the absence of the main glycosyltransferase pbp2 (Wang et al., 2001; Reed et al., 2015). Collectively, this data revealed that the *tcaA* inactivation altered the expression of cell wall-associated genes, probably allowing the cell wall to better withstand external pressures, and it would be interesting to explore *glyS* and *sgtB* involvement in cell wall biogenesis during glycopeptide treatment or in the persister cell formation.

## 4. Discussion

Glycopeptide resistance in MRSA is a global problem and vancomycin- and teicoplanin-resistant strains are of major clinical relevance. Several studies have reported the emergence of teicoplanin and vancomycin resistance in *S. aureus* (Wang Y. et al., 2017; Szymanek-Majchrzak et al., 2018; Wu et al., 2021). Typically, teicoplanin and vancomycin bind to D-alanine-D-alanine subunits of the murein monomer and cross resistance could develop between teicoplanin and vancomycin (Bakthavatchalam et al., 2019). The cell wall thickness also contributed to the development of vancomycin and teicoplanin resistance in *S. aureus* (Cui et al., 2006; Bakthavatchalam et al., 2019). Two-component systems, such as walKR, vraSR, graSR, and tcaRAB operon, are linked to the development of teicoplanin and vancomycin resistance in *S. aureus* (Howden et al., 2010). Also, *vanA* and *tcaA* are considered to give resistance to vancomycin and teicoplanin in *S. aureus* (Chang et al., 2003; Maki et al., 2004), while a report also claimed that the effects of the *tcaA* deletion on resistance were strain specific because the *tcaA* mutant in the *S. aureus* Col strain had higher MIC than the *tcaA* mutant in the BB1372 strain (Brandenberger et al., 2000). *tcaA* has been reported to be upregulated by vancomycin (Kuroda et al., 2003), oxacillin, and teicoplanin treatment (Utaida et al., 2003).The *tcaA* gives strong resistance when inactivated while its overexpression from an inducible promoter was effective in lowering teicoplanin resistance (Maki et al., 2004). A study reported that pbp2 and pbp4 lead to *S. aureus* cell wall thickening that reduced vancomycin susceptibility (Sieradzki and Tomasz, 2003). The pbp2 upregulation promoted cell wall synthesis, while pbp4 downregulation resulted in a decrease in murein cross-linking that increased D-alanine-D-alanine production (Gardete and Tomasz, 2014). Importantly, mutations in different genes such as *yvqF* (Elsaghier et al., 2002), *vraSR* (Yoo et al., 2013), and *rpoB* were also associated with teicoplanin and vancomycin resistance (Watanabe et al., 2011). In the present investigation, we detected higher MICs of 16 μg/mL and 10 μg/mL for teicoplanin and vancomycin, respectively, which is in accordance with previous reports where teicoplanin and vancomycin MICs of 16 μg/mL and 2 μg/mL were reported for resistant isolates (Wang Y. et al., 2017). Previously, it was shown that the overexpression of *tcaA* in clinical strains decreased glycopeptide MICs while its inactivation resulted in glycopeptide resistance (Maki et al., 2004). Here, the data revealed that *S. aureus* suppressed the *tcaA* expression under the influence of COVID-19 infection and developed glycopeptide resistance. The results were derived from RNA-seq, RT-qPCR, and Western blot analysis that disclosed *tcaA* suppression and inactivation. The results were corroborated by creating a *tcaA* mutant which revealed that *tcaA* is solely responsible for the emergence of glycopeptide resistance and persistence. The *tcaA* persistence assay revealed a 10–11 fold and 13–14 fold increase in persister cells toward vancomycin and teicoplanin treatment, respectively. The *tcaA*-dependent persisters were not detected in azithromycin and ciprofloxacin challenge assays that indicated persisters might be dependent on specific gene function or dysfunction in a specific environment. We also confirmed that *tcaA* deletion did not affect azithromycin resistance whereas vancomycin and teicoplanin susceptibility was decreased as previously reported (Brandenberger et al., 2000; Maki et al., 2004). The study of Vogwill et al. (2016) investigated the coevolution of resistance and persistence to ciprofloxacin and rifampicin across the genus *Pseudomonas* and concluded that persistence correlates positively to antibiotic resistance across *Pseudomonas* strains (Vogwill et al., 2016). From theoretical and experimental analysis, Windels et al. (2019) proposed that persisters facilitated genetic resistance and increased survival and mutation rates that might affect the evolution of clinical resistance in *E. coli* (Windels et al., 2019). To date, *S. aureus* persisters increased with the decrease in intracellular ATP (Conlon et al., 2016), while decreasing upon deletion of the msaABCR operon (Pandey et al., 2021). Moreover, phenol-soluble modulin toxins expression reduced persisters in *S. aureus* (Baldry et al., 2020), and the expression of T7SS facilitated *S. aureus* survival in persistent infection. *S. aureus* T7SS is crucial for its virulence and the *essC* is thought to be a central membrane transporter (Burts et al., 2005; Jäger et al., 2018). Studies have shown that deletion of the entire T7SS or its components (EsxA, EssC, EsxB, EsaD, etc.) decreased *S. aureus* virulence (Burts et al., 2005, 2008; Anderson et al., 2017). EsxA and EsxB are the secretory proteins of T7SS and are required for establishing *S. aureus* infection in the host and the *EsxAB* mutants caused a decrease in abscess formation in mice (Burts et al., 2005). Currently, a high expression of *EsxAB* and a low expression of *essC* were detected that might facilitate *S. aureus* survival during host infection, however, the mechanism behind this phenomenon remained elusive. Even though the *EsxAB* genes are involved in *S. aureus* persistence and *essC* is required for establishing *S. aureus* infection during lung infection (Ishii et al., 2014), details of their altered chemistry in host infection need further investigation. From current data, we conclude that *tcaA* suppression and deletion induced persistence in *S. aureus*, and *glyS* and *sgtB* showed increased expression in the *tcaA* mutant strain that might indicate a cumulative approach toward cell wall protection. Although *glyS* and *sgtB* roles in cell wall synthesis in *S. aureus* are well defined (Wang et al., 2001; Schneider et al., 2004; Giannouli et al., 2009; Reed et al., 2015), their involvement in cell wall protection during host infection and persister cell formation remained obscure. Altogether, this is the first study to reveal persister cell formation due to *tcaA* inactivation, and conclude that the emergence of resistance might reflect the adaptation mechanism of persister cell genotype in future.

## 5. Conclusion

It is well known that *S. aureus* can change its fitness during infection to increase its survival in a hostile environment. In the present study, *S. aureus,* showing an intermediate level of glycopeptide resistance, was recovered from a COVID-19 patient. Genome analysis revealed a high expression of genes involved in cell wall biosynthesis and a low expression of virulence regulatory genes. The data summarize that *tcaA* inactivation gave rise to persisters that tolerated a high concentration of glycopeptides and resuscitated the bacterial population. This showed the involvement of genetic determinants in the development of persisters. Here, we conclude that if bacterial genes remain the main culprit of persister cell formation, then there would be concern regarding the potential spread of the persistence-associated genes. Although more remains to be explored concerning the genetic basis of persisters, our data will increase the understanding of the mechanism of persister cell formation during host infection.

## Data availability statement

The original contributions presented in the study are included in the article/Supplementary material, further inquiries can be directed to the corresponding authors.

## Author contributions

GH and HG: conceptualization and writing-original draft. GH, HG, MR, and AH: data curation and formal analysis. GH, HG, HE, PA, and IM: methodology and software. GH, MR, PA, HE, and IM: resources and funding acquisition. HG, MR and AH: supervision and project administration: AH, MR, HG, PA, HE, and IM: review and editing. All authors proofread the article and approved the current version.

## Footnotes

^1^http://www.genome.jp/kegg/

## References

[ref1] Anders S. Huber W. (2010). Differential expression analysis for sequence count data. Nat. Prec.:1. doi: 10.1038/npre.2010.4282.2 PMC3218662 20979621

[ref2] Anderson M. Ohr R. J. Aly K. A. Nocadello S. Kim H. K. Schneewind C. E. (2017). EssE promotes *Staphylococcus aureus* ESS-dependent protein secretion to modify host immune responses during infection. J. Bacteriol. 199:e00527-16. doi: 10.1128/JB.00527-16 27795322 PMC5165088

[ref3] Appelbaum P. (2006). The emergence of vancomycin-intermediate and vancomycin-resistant *Staphylococcus aureus*. Clin. Microbiol. Infect. 12, 16–23. doi: 10.1111/j.1469-0691.2006.01344.x 16445720

[ref4] Aqib A. Rodriguez-Morales A.J. (2021). Insights into drug resistance in Staphylococcus aureus. London. IntechOpen.

[ref5] Bae T. Schneewind O. (2006). Allelic replacement in *Staphylococcus aureus* with inducible counter-selection. Plasmid 55, 58–63. doi: 10.1016/j.plasmid.2005.05.005, PMID: 16051359

[ref6] Bakthavatchalam Y. D. Babu P. Munusamy E. Dwarakanathan H. T. Rupali P. Zervos M.. (2019). Genomic insights on heterogeneous resistance to vancomycin and teicoplanin in methicillin-resistant *Staphylococcus aureus*: a first report from South India. PLoS One 14:e0227009. doi: 10.1371/journal.pone.0227009, PMID: 31887179 PMC6936811

[ref7] Baldry M. Bojer M. S. Najarzadeh Z. Vestergaard M. Meyer R. L. Otzen D. E.. (2020). Phenol-soluble modulins modulate persister cell formation in *Staphylococcus aureus*. Front. Microbiol. 11:573253. doi: 10.3389/fmicb.2020.573253, PMID: 33240231 PMC7680730

[ref8] Bao Y. Li Y. Jiang Q. Zhao L. Xue T. Hu B.. (2013). Methylthioadenosine/S-adenosylhomocysteine nucleosidase (Pfs) of *Staphylococcus aureus* is essential for the virulence independent of LuxS/AI-2 system. Int. J. Med. Microbiol. 303, 190–200. doi: 10.1016/j.ijmm.2013.03.004, PMID: 23611628

[ref9] Blair J. M. Webber M. A. Baylay A. J. Ogbolu D. O. Piddock L. J. (2015). Molecular mechanisms of antibiotic resistance. Nat. Rev. Microbiol. 13, 42–51. doi: 10.1038/nrmicro3380 25435309

[ref10] Brandenberger M. Tschierske M. Giachino P. Wada A. Berger-Bächi B. (2000). Inactivation of a novel three-cistronic operon tcaR-tcaA-tcaB increases teicoplanin resistance in *Staphylococcus aureus*. Biochim. Biophys. Acta 1523, 135–139. doi: 10.1016/S0304-4165(00)00133-1, PMID: 11042376

[ref11] Brauner A. Fridman O. Gefen O. Balaban N. Q. (2016). Distinguishing between resistance, tolerance and persistence to antibiotic treatment. Nat. Rev. Microbiol. 14, 320–330. doi: 10.1038/nrmicro.2016.34, PMID: 27080241

[ref12] Brückner R. (1997). Gene replacement in Staphylococcus carnosus and *Staphylococcus xylosus*. FEMS Microbiol. Lett. 151, 1–8. doi: 10.1016/S0378-1097(97)00116-x, PMID: 9198277

[ref13] Brunet F. Vedel G. Dreyfus F. Vaxelaire J. Giraud T. Schremmer B. (1990). Failure of teicoplanin therapy in two neutropenic patients with staphylococcal septicemia who recovered after administration of vancomycin. Eur. J. Clin. Microbiol. Infect. Dis. 9, 145–147. doi: 10.1007/BF01963643, PMID: 2138543

[ref14] Burts M. L. DeDent A. C. Missiakas D. M. (2008). EsaC substrate for the ESAT-6 secretion pathway and its role in persistent infections of *Staphylococcus aureus*. Mol. Microbiol. 69, 736–746. doi: 10.1111/j.1365-2958.2008.06324.x, PMID: 18554323 PMC2597432

[ref15] Burts M. L. Williams W. A. DeBord K. Missiakas D. M. (2005). EsxA and EsxB are secreted by an ESAT-6-like system that is required for the pathogenesis of *Staphylococcus aureus* infections. Proc. Natl. Acad. Sci. 102, 1169–1174. doi: 10.1073/pnas.0405620102, PMID: 15657139 PMC545836

[ref16] Cameron D. R. Shan Y. Zalis E. A. Isabella V. Lewis K. (2018). A genetic determinant of persister cell formation in bacterial pathogens. J. Bacteriol. 200:e00303-18. doi: 10.1128/JB.00303-18 29941425 PMC6088157

[ref17] Chang S. Sievert D. M. Hageman J. C. Boulton M. L. Tenover F. C. Downes F. P.. (2003). Infection with vancomycin-resistant *Staphylococcus aureus* containing the vanA resistance gene. N. Engl. J. Med. 348, 1342–1347. doi: 10.1056/NEJMoa025025 12672861

[ref18] Chen K.-Y. Chang H.-J. Hsu P.-C. Yang C.-C. Chia J.-H. Wu T.-L.. (2013). Relationship of teicoplanin MICs to treatment failure in teicoplanin-treated patients with methicillin-resistant *Staphylococcus aureus* pneumonia. J. Microbiol. Immunol. Infect. 46, 210–216. doi: 10.1016/j.jmii.2012.06.010, PMID: 22999099

[ref19] Clinical and Laboratory Standards Institute (2018). “Performance standards for antimicrobial susceptibility testing” in CLSI supplement M100. 28th edn. (Wayne, PA, USA: Clinical and Laboratory Standards Institute). Available at: https://clsi.org/media/1930/m100ed28_sample.pdf

[ref20] Conlon B. P. Rowe S. E. Gandt A. B. Nuxoll A. S. Donegan N. P. Zalis E. A.. (2016). Persister formation in *Staphylococcus aureus* is associated with ATP depletion. Nat. Microbiol. 1, 1–7. doi: 10.1038/nmicrobiol.2016.51 PMC4932909 27572649

[ref21] Corrigan R. M. Foster T. J. (2009). An improved tetracycline-inducible expression vector for *Staphylococcus aureus*. Plasmid 61, 126–129. doi: 10.1016/j.plasmid.2008.10.001, PMID: 18996145

[ref22] Cui L. Iwamoto A. Lian J.-Q. Neoh H. M. Maruyama T. Horikawa Y. (2006). Novel mechanism of antibiotic resistance originating in vancomycin-intermediate *Staphylococcus aureus*. Antimicrob. agents chemother. 50, 428–438. doi: 10.1128/AAC.50.2.428-438.2006, PMID: 16436693 PMC1366884

[ref23] Eisenreich W. Rudel T. Heesemann J. Goebel W. (2021). Persistence of intracellular bacterial pathogens—with a focus on the metabolic perspective. Front. Cell. Infect. Microbiol. 10:615450. doi: 10.3389/fcimb.2020.615450, PMID: 33520740 PMC7841308

[ref24] Elsaghier A. A. Aucken H. M. Hamilton-Miller J. M. Shaw S. Kibbler C. C. (2002). Resistance to teicoplanin developing during treatment of methicillin-resistant *Staphylococcus aureus* infection. J. Antimicrob. Chemother. 49, 423–424. doi: 10.1093/jac/49.2.423 11815594

[ref25] Gardete S. Tomasz A. (2014). Mechanisms of vancomycin resistance in *Staphylococcus aureus*. J. Clin. Invest. 124, 2836–2840. doi: 10.1172/JCI68834, PMID: 24983424 PMC4071404

[ref26] Ghanizadeh A. Najafizade M. Rashki S. Marzhoseyni Z. Motallebi M. (2021). Genetic diversity, antimicrobial resistance pattern, and biofilm formation in *Klebsiella pneumoniae* isolated from patients with coronavirus disease 2019 (COVID-19) and ventilator-associated pneumonia. Biomed. Res. Int. 2021, 1–11. doi: 10.1155/2021/2347872, PMID: 34957300 PMC8703158

[ref27] Giannouli S. Kyritsis A. Malissovas N. Becker H. D. Stathopoulos C. (2009). On the role of an unusual tRNAGly isoacceptor in *Staphylococcus aureus*. Biochimie 91, 344–351. doi: 10.1016/j.biochi.2008.10.009, PMID: 19014993

[ref28] Habib G. Mahmood K. Ahmad L. Gul H. Hayat A. Rehman M. U. (2023). Clinical manifestations of active tuberculosis patients coinfected with severe acute respiratory syndrome coronavirus-2. J. Clin. Tuberc. Other Mycobact. Dis. 31:100359. doi: 10.1016/j.jctube.2023.100359, PMID: 36945658 PMC9985913

[ref29] Habib G. Mahmood K. Gul H. Tariq M. Ain Q. U. Hayat A.. (2022). Pathophysiology of methicillin-resistant *Staphylococcus aureus* superinfection in COVID-19 patients. Pathophysiology 29, 405–413. doi: 10.3390/pathophysiology29030032, PMID: 35997388 PMC9397082

[ref30] Habib G. Zhu J. Sun B. (2020). A novel type I toxin-antitoxin system modulates persister cell formation in *Staphylococcus aureus*. Int. J. Med. Microbiol. 310:151400. doi: 10.1016/j.ijmm.2020.151400, PMID: 32001143

[ref31] Handwerger S. Tomasz A. (1985). Antibiotic tolerance among clinical isolates of bacteria. Annu. Rev. Pharmacol. Toxicol. 25, 349–380. doi: 10.1146/annurev.pa.25.040185.002025 3890707

[ref32] Hiramatsu K. (2001). Vancomycin-resistant *Staphylococcus aureus*: a new model of antibiotic resistance. Lancet Infect. Dis. 1, 147–155. doi: 10.1016/S1473-3099(01)00091-3 11871491

[ref33] Howden B. P. Davies J. K. Johnson P. D. Stinear T. P. Grayson M. L. (2010). Reduced vancomycin susceptibility in *Staphylococcus aureus*, including vancomycin-intermediate and heterogeneous vancomycin-intermediate strains: resistance mechanisms, laboratory detection, and clinical implications. Clin. Microbiol. Rev. 23, 99–139. doi: 10.1128/CMR.00042-09, PMID: 20065327 PMC2806658

[ref34] Howden B. P. Stinear T. P. Allen D. L. Johnson P. D. Ward P. B. Davies J. K. (2008). Genomic analysis reveals a point mutation in the two-component sensor gene graS that leads to intermediate vancomycin resistance in clinical *Staphylococcus aureus*. Antimicrob. Agents Chemother. 52, 3755–3762. doi: 10.1128/AAC.01613-07, PMID: 18644967 PMC2565880

[ref35] Hu J. Zhang X. Liu X. Chen C. Sun B. (2015). Mechanism of reduced vancomycin susceptibility conferred by walK mutation in community-acquired methicillin-resistant *Staphylococcus aureus* strain MW2. Antimicrob. Agents Chemother. 59, 1352–1355. doi: 10.1128/AAC.04290-14, PMID: 25451044 PMC4335861

[ref36] Hu Q. Peng H. Rao X. (2016). Molecular events for promotion of vancomycin resistance in vancomycin intermediate *Staphylococcus aureus*. Front. Microbiol. 7:1601. doi: 10.3389/fmicb.2016.01601, PMID: 27790199 PMC5062060

[ref37] Ishii K. Adachi T. Yasukawa J. Suzuki Y. Hamamoto H. Sekimizu K. (2014). Induction of virulence gene expression in *Staphylococcus aureus* by pulmonary surfactant. Infect. immun. 82, 1500–1510. doi: 10.1128/IAI.01635-13, PMID: 24452679 PMC3993393

[ref38] Jäger F. Kneuper H. Palmer T. (2018). EssC is a specificity determinant for *Staphylococcus aureus* type VII secretion. Microbiology 164, 816–820. doi: 10.1099/mic.0.000650, PMID: 29620499 PMC5994694

[ref39] Jia G. Rao Z. Zhang J. Li Z. Chen F. (2013). Tetraether biomarker records from a loess-paleosol sequence in the western Chinese loess plateau. Front. Microbiol. 4:199. doi: 10.3389/fmicb.2013.00199 23898324 PMC3710990

[ref40] Kanehisa M. Araki M. Goto S. Hattori M. Hirakawa M. Itoh M.. (2007). KEGG for linking genomes to life and the environment. Nucleic Acids Res. 36, D480–D484. doi: 10.1093/nar/gkm882, PMID: 18077471 PMC2238879

[ref41] Kuroda M. Kuroda H. Oshima T. Takeuchi F. Mori H. Hiramatsu K. (2003). Two-component system VraSR positively modulates the regulation of cell-wall biosynthesis pathway in *Staphylococcus aureus*. Mol. Microbiol. 49, 807–821. doi: 10.1046/j.1365-2958.2003.03599.x, PMID: 12864861

[ref42] Levin-Reisman I. Ronin I. Gefen O. Braniss I. Shoresh N. Balaban N. Q. (2017). Antibiotic tolerance facilitates the evolution of resistance. Science 355, 826–830. doi: 10.1126/science.aaj2191 28183996

[ref43] Levin B. R. McCall I. C. Perrot V. Weiss H. Ovesepian A. Baquero F. (2017). A numbers game: ribosome densities, bacterial growth, and antibiotic-mediated stasis and death. MBio 8:e02253-16. doi: 10.1128/mBio.02253-16 28174311 PMC5296603

[ref44] Lewis K. (2010). Persister cells. Annu. Rev. Microbiol. 64, 357–372. doi: 10.1146/annurev.micro.112408.134306 20528688

[ref45] Lin L. Wang X. Wang W. Zhou X. Hargreaves J. R. (2020). Cleaning up China’s medical cabinet—an antibiotic take-back programme to reduce household antibiotic storage for unsupervised use in rural China: a mixed-methods feasibility study. Antibiotics 9:212. doi: 10.3390/antibiotics9050212, PMID: 32349422 PMC7277206

[ref46] Maki H. McCallum N. Bischoff M. Wada A. Berger-Bächi B. (2004). tcaA inactivation increases glycopeptide resistance in *Staphylococcus aureus*. Antimicrob. agents chemother. 48, 1953–1959. doi: 10.1128/AAC.48.6.1953-1959.2004, PMID: 15155184 PMC415614

[ref47] Mao X. Cai T. Olyarchuk J. G. Wei L. (2005). Automated genome annotation and pathway identification using the KEGG Orthology (KO) as a controlled vocabulary. Bioinformatics 21, 3787–3793. doi: 10.1093/bioinformatics/bti430, PMID: 15817693

[ref48] Mohiuddin S. G. Ghosh S. Ngo H. G. Sensenbach S. Karki P. Dewangan N. K.. (2021). Cellular self-digestion and persistence in Bacteria. Microorganisms 9:2269. doi: 10.3390/microorganisms9112269, PMID: 34835393 PMC8626048

[ref49] Pandey S. Sahukhal G. S. Elasri M. O. (2021). The msaABCR operon regulates persister formation by modulating energy metabolism in *Staphylococcus aureus*. Front. Microbiol. 12:657753. doi: 10.3389/fmicb.2021.657753, PMID: 33936014 PMC8079656

[ref50] Personnic N. Doublet P. Jarraud S. (2023). Intracellular persister: a stealth agent recalcitrant to antibiotics. Front. Cell. Infect. Microbiol. 13:1141868. doi: 10.3389/fcimb.2023.1141868, PMID: 37065203 PMC10102521

[ref51] Reed P. Atilano M. L. Alves R. Hoiczyk E. Sher X. Reichmann N. T.. (2015). *Staphylococcus aureus* survives with a minimal peptidoglycan synthesis machine but sacrifices virulence and antibiotic resistance. PLoS Pathog. 11:e1004891. doi: 10.1371/journal.ppat.1004891, PMID: 25951442 PMC4423922

[ref52] Renzoni A. Kelley W. L. Barras C. Monod A. Huggler E. François P. (2009). Identification by genomic and genetic analysis of two new genes playing a key role in intermediate glycopeptide resistance in *Staphylococcus aureus*. Antimicrob. Agents Chemother. 53, 903–911. doi: 10.1128/AAC.01287-08, PMID: 19104009 PMC2650575

[ref53] Schneider T. Senn M. M. Berger-Bächi B. Tossi A. Sahl H. G. Wiedemann I. (2004). In vitro assembly of a complete, pentaglycine interpeptide bridge containing cell wall precursor (lipid II-Gly5) of *Staphylococcus aureus*. Mol. Microbiol. 53, 675–685. doi: 10.1111/j.1365-2958.2004.04149.x, PMID: 15228543

[ref54] Schulthess B. Meier S. Homerova D. Goerke C. Wolz C. Kormanec J.. (2009). Functional characterization of the σB-dependent yabJ-spoVG operon in *Staphylococcus aureus*: role in methicillin and glycopeptide resistance. Antimicrob. Agents Chemother. 53, 1832–1839. doi: 10.1128/AAC.01255-08, PMID: 19223635 PMC2681525

[ref55] Shang Y. Wang X. Chen Z. Lyu Z. Lin Z. Zheng J.. (2020). *Staphylococcus aureus* PhoU homologs regulate persister formation and virulence. Front. Microbiol. 11:865. doi: 10.3389/fmicb.2020.00865, PMID: 32670206 PMC7326077

[ref56] Shariati A. Dadashi M. Moghadam M. T. van Belkum A. Yaslianifard S. Darban-Sarokhalil D. (2020). Global prevalence and distribution of vancomycin resistant, vancomycin intermediate and heterogeneously vancomycin intermediate *Staphylococcus aureus* clinical isolates: a systematic review and meta-analysis. Sci. Rep. 10:12689. doi: 10.1038/s41598-020-69058-z, PMID: 32728110 PMC7391782

[ref57] Sieradzki K. Tomasz A. (2003). Alterations of Cell Wall structure and MetabolismAccompany reduced susceptibility to vancomycin in an IsogenicSeries of clinical isolates of Staphylococcusaureus. J. Bacteriol. 185, 7103–7110. doi: 10.1128/JB.185.24.7103-7110.2003, PMID: 14645269 PMC296238

[ref58] Sieradzki K. Villari P. Tomasz A. (1998). Decreased susceptibilities to teicoplanin and vancomycin among coagulase-negative methicillin-resistant clinical isolates of staphylococci. Antimicrob. Agents Chemother. 42, 100–107. doi: 10.1128/AAC.42.1.100, PMID: 9449268 PMC105463

[ref59] Song K.-H. Kim M. Kim C. J. Cho J. E. Choi Y. J. Park J. S.. (2017). Impact of vancomycin MIC on treatment outcomes in invasive *Staphylococcus aureus* infections. Antimicrob. Agents Chemother. 61, e01845–e01816. doi: 10.1128/AAC.01845-16 27956430 PMC5328555

[ref60] Szymanek-Majchrzak K. Mlynarczyk A. Mlynarczyk G. (2018). Characteristics of glycopeptide-resistant *Staphylococcus aureus* strains isolated from inpatients of three teaching hospitals in Warsaw, Poland. Antimicrob. Resist. Infect. Control 7, 1–6. doi: 10.1186/s13756-018-0397-y 30181870 PMC6114487

[ref61] Tariq F. N. Shafiq M. Khawar N. Habib G. Gul H. Hayat A.. (2023). The functional repertoire of AmpR in the AmpC β-lactamase high expression and decreasing β-lactam and aminoglycosides resistance in ESBL *Citrobacter freundii*. Heliyon 9:e19486. doi: 10.1016/j.heliyon.2023.e19486, PMID: 37662790 PMC10472055

[ref62] Utaida S. Dunman P. Macapagal D. Murphy E. Projan S. Singh V. (2003). Genome-wide transcriptional profiling of the response of *Staphylococcus aureus* to cell-wall-active antibiotics reveals a cell-wall-stress stimulon. Microbiology 149, 2719–2732. doi: 10.1099/mic.0.26426-0, PMID: 14523105

[ref63] Valihrach L. Demnerova K. (2012). Impact of normalization method on experimental outcome using RT-qPCR in *Staphylococcus aureus*. J. Microbiol. Methods 90, 214–216. doi: 10.1016/j.mimet.2012.05.008, PMID: 22613804

[ref64] Vogwill T. Comfort A. Furió V. MacLean R. (2016). Persistence and resistance as complementary bacterial adaptations to antibiotics. J. Evol. Biol. 29, 1223–1233. doi: 10.1111/jeb.12864, PMID: 26999656 PMC5021160

[ref65] Wang Q. Lu F. Lan R. (2017). RNA-sequencing dissects the transcriptome of polyploid cancer cells that are resistant to combined treatments of cisplatin with paclitaxel and docetaxel. Mol. BioSyst. 13, 2125–2134. doi: 10.1039/C7MB00334J, PMID: 28825433

[ref66] Wang Q. M. Peery R. B. Johnson R. B. Alborn W. E. Yeh W.-K. Skatrud P. L. (2001). Identification and characterization of a monofunctional glycosyltransferase from *Staphylococcus aureus*. J. Bacteriol. 183, 4779–4785. doi: 10.1128/JB.183.16.4779-4785.2001, PMID: 11466281 PMC99532

[ref67] Wang W. Chen J. Chen G. Du X. Cui P. Wu J. (2015). Transposon mutagenesis identifies novel genes associated with *Staphylococcus aureus* persister formation. Front. Microbiol. 6:1437. doi: 10.3389/fmicb.2015.01437 26779120 PMC4689057

[ref68] Wang Y. Bojer M. S. George S. E. Wang Z. Jensen P. R. Wolz C.. (2018). Inactivation of TCA cycle enhances *Staphylococcus aureus* persister cell formation in stationary phase. Sci. Rep. 8:10849. doi: 10.1038/s41598-018-29123-0, PMID: 30022089 PMC6052003

[ref69] Wang Y. Li X. Jiang L. Han W. Xie X. Jin Y.. (2017). Novel mutation sites in the development of vancomycin-intermediate resistance in *Staphylococcus aureus*. Front. Microbiol. 7:2163. doi: 10.3389/fmicb.2016.02163 28119680 PMC5222870

[ref70] Watanabe Y. Cui L. Katayama Y. Kozue K. Hiramatsu K. (2011). Impact of rpoB mutations on reduced vancomycin susceptibility in *Staphylococcus aureus*. J. Clin. Microbiol. 49, 2680–2684. doi: 10.1128/JCM.02144-10, PMID: 21525224 PMC3147882

[ref71] Windels E. M. Michiels J. E. Fauvart M. Wenseleers T. Van den Bergh B. Michiels J. (2019). Bacterial persistence promotes the evolution of antibiotic resistance by increasing survival and mutation rates. ISME J. 13, 1239–1251. doi: 10.1038/s41396-019-0344-9, PMID: 30647458 PMC6474225

[ref72] Wu Q. Sabokroo N. Wang Y. Hashemian M. Karamollahi S. Kouhsari E. (2021). Systematic review and meta-analysis of the epidemiology of vancomycin-resistance *Staphylococcus aureus* isolates. Antimicrob. Resist. Infect. Control 10, 1–13. doi: 10.1186/s13756-021-00967-y 34193295 PMC8247230

[ref73] Yoo J. I. Kim J. W. Kang G. S. Kim H. S. Yoo J. S. Lee Y. S. (2013). Prevalence of amino acid changes in the yvqF, vraSR, graSR, and tcaRAB genes from vancomycin intermediate resistant *Staphylococcus aureus*. J. Microbiol. 51, 160–165. doi: 10.1007/s12275-013-3088-7, PMID: 23625215

[ref74] Young M. D. Wakefield M. J. Smyth G. K. Oshlack A. (2010). Gene ontology analysis for RNA-seq: accounting for selection bias. Genome Biol. 11, R14–R12. doi: 10.1186/gb-2010-11-2-r14 20132535 PMC2872874

[ref75] Zhao C. Shu X. Sun B. (2017). Construction of a gene knockdown system based on catalytically inactive (“dead”) Cas9 (dCas9) in *Staphylococcus aureus*. Appl. Environ. Microbiol. 83, e00291–e00217. doi: 10.1128/AEM.00291-17 28411216 PMC5452804

