# Supplementary figures and images for: Teicoplanin associated gene tcaA inactivation increases persister cell formation in Staphylococcus aureus
Source: Front Microbiol. 2023 Oct 13;14:1241995. doi: 10.3389/fmicb.2023.1241995 (PMC10611510; doi:10.3389/fmicb.2023.1241995)

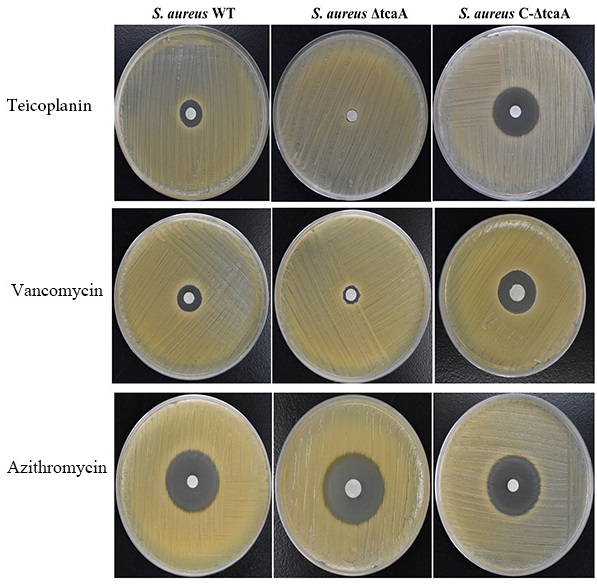

Supplement: Supplementary file 1 [file Image_1.JPEG]
